# Reservoir inventory for China in 2016 and 2021

**DOI:** 10.1038/s41597-023-02515-2

**Published:** 2023-09-09

**Authors:** Haiying Zhang, Fang Chen, Lei Wang, Ning Wang, Bo Yu

**Affiliations:** 1 International Research Center of Big Data for Sustainable Development Goals, Beijing, 100094 China; 2 Key Laboratory of Digital Earth Science, Aerospace Information Research Institute, Chinese Academy of Sciences, Beijing, 100094 China; 3 University of Chinese Academy of Sciences, Beijing, 100049 China; 4 School of Computer Science and Information Security, Guilin University of Electronic Technology, Guilin, 541004 China; 5 College of Urban and Environmental Sciences, Peking University, Beijing, 100871 China

**Keywords:** Hydrology, Geography

## Abstract

Reservoir inventories are essential for investigating the impact of climate change and anthropogenic activities on water scape changes. They provide fundamental data sources to explore the sustainability and management efficiency of water resources. However, publicly released reservoir inventories are currently limited to a single temporal domain. As a result, the effectiveness of governmental policy implementation on water resources remains to be explored due to the lack of multi-time datasets. In this study, we generated a reservoir inventory for China for the years 2016 and 2021 with an overall accuracy of 99.71%. The reservoirs were visually interpreted from annually composited Landsat images, and each reservoir is represented by a polygon with attributes of reservoir name, area and storage capacity. About 10.32% of the reservoirs have increased storage capacity from 2016 to 2021, while 22.73% have decreased. Most provinces and river basins in China have expanded their accumulated storage capacity from 2016 to 2021.

## Background & Summary

Reservoirs are recognized as one of the most effective means to deal with agricultural irrigation, hydropower generation, water supply, daily recreation, flood control and disaster relief^1–3^. To make the best use of water resources and meet the continuous demands of water consumption and hydropower, numerous reservoirs have been constructed worldwide^4–6^. One fifth of the global cultivated land is used for irrigation to provide 33% of the world’s food^7^. Recent research suggests that by 2050, 70% more food will be needed to accommodate shifts in dietary patterns and the 40% increase in the world’s population^8^. The additional food will be partly produced from irrigation, requiring 11% more water from reservoirs^2^. Moreover, approximately 20% of the world’s electricity is generated by hydropower, which amounts to 7% of worldwide energy^2^. China is an arid country that seriously lacks water. The total freshwater resource of China accounts for only 6% of the world’s total resources, but the average water resource capacity per capita is only one fourth of the global average level^9^. Moreover, China is the country that consumes the largest amount of water^10^. As a result, China has built numerous reservoirs over the past few decades to meet the increasing demands for effective water use, flood control, and power generation^11–14^. In the 1950s, there were only 20 reservoirs with a storage capacity greater than 0.1 cubic kilometers^3^. By 2019, 98,112 reservoirs with a total capacity of 898.3 billion km^3^ had been constructed^15^.

To deal with the severe pressure from the increasing population and anthropogenic impacted droughts in the future, it is crucial to estimate water resource availability in terms of surface area, storage capacity, and spatial distribution for effective management strategy proposal^16^. The development of remote sensing technology made it possible to delineate georeferenced reservoirs with detailed area and storage capacity from remotely sensed images^17,18^. Numerous research studies have delineated reservoirs from multi-source remotely sensed images to analyze the impact of anthropogenic activities and create global or regional datasets of reservoirs^2,7,19,20^. Several dam and reservoir datasets have been proposed, such as the World Register of Dams (WRD)^21^, the Global GeOreferenced Database of Dams (GOODD) V1^22^, and the Global Reservoir and Dam database (GRanD)^2^. However, the datasets generally cover limited records, and the reservoir boundaries interpreted lack spatial resolution consistency. Some large reservoirs have coarse digitized boundaries while some small reservoirs have detailed boundaries due to the multiple spatial resolutions of images used for interpretation. Moreover, most records in the datasets lack detailed reservoir information, including area and storage capacity. That hinders thorough and comprehensive analysis in the absence of detailed reservoir distribution patterns^23^.

Landsat images have been widely used to delineate reservoirs in various locations, such as Zimbabwe^24^, India^25^, Ghana^26^, the Yellow River of China^27^, and mainland China^7^. There have been many reservoir datasets proposed based on Landsat images due to their global coverage and public availability, with a spatial resolution of 30 m. Application of the Google Earth Engine (GEE) platform has made it possible to map reservoirs for large-scale areas^28^. Wang, *et al*. delineated large dams, reservoirs, and lakes of China based on spectral indices calculated from Landsat images obtained from GEE in 2019^29^. Meanwhile, Song, *et al*. presented a comprehensive reservoir inventory dataset of China Reservoir Dataset (CRD) by compiling multiple public reservoir datasets constructed from GEE^6^. Although the published reservoir datasets cover detailed distribution patterns of a single temporal phase, they lack the variation patterns of reservoirs in different time domains, which make it difficult to analyze the sustainability improvement of water use in different time domains.

In 2016, the Chinese government released regulations on Farmland Water Conservancy to encourage the use of reservoirs for irrigation. Medium and large reservoirs are significant in agricultural irrigation. Although medium and large reservoirs in China account for less than 5% of total reservoirs, the corresponding storage capacity accounts for 92%. However, with the long-term use, some reservoirs have been dilapidated mainly because of lacking reinforcement^30^. In 2021, the Chinese National Development and Reform Commission announced to double the budget to reinforce the hazardous reservoir to 4 billion. Before implementing the policy in strengthening the hazardous reservoirs with greater effort, the effectiveness of the policy in encouraging the use of reservoirs for irrigation remains to be explored through recent technical developments. However, analyzing the patterns of reservoir storage capacity change with irrigation cropland area between 2016 and 2021 from the China Water Resources Statistical Yearbook, released by the Chinese government, is challenging without detailed reservoir inventory in the two time-domains. Local investigation is the most commonly used method, but it is costly and laborious. Exploring the distribution pattern change of medium and large reservoirs through publicly available remotely sensed images in China can generally delineate the water resources utilization status change from year 2016 to 2021.

Aiming to propose a reservoir inventory of China for 2016 and 2021 to explore the reservoir storage capacity change of medium and large reservoirs and evaluate the change in response to the regulation of Farmland Water Conservancy, we generated a reservoir inventory dataset (MLRC) over China for 2016 and 2021 by classification based on annually composited Landsat images from GEE, aided by geological datasets of the National 1:250,000 Public Basic Geographic Database of 2019, and reservoir locations acquired from Baidu Maps in 2016 and 2021. We composited the Landsat images annually as base maps for potential reservoir extraction and validation through visual interpretation. The locations of reservoirs in Geological datasets and Baidu Maps are used to initialize the reservoirs for the sake of visual interpretation. The reservoir inventories were evaluated by stratified random sampling^31^, referring to higher spatial resolution images from Google Earth. Our shared dataset consists of a detailed reservoir inventory with area and storage capacity. Based on the reservoir inventories for 2 years, we explored the storage capacity change of medium and large reservoirs, together with the irrigation cropland area change, aided by publicly available 30 m spatial resolution land cover land use products^32^. This study not only evaluates the reservoir capacity change for water conservancy but also advances research in other water resource utilization-related research by providing reservoir inventories in two different time domains.

## Methods

### Study area

**Fig. 1:**
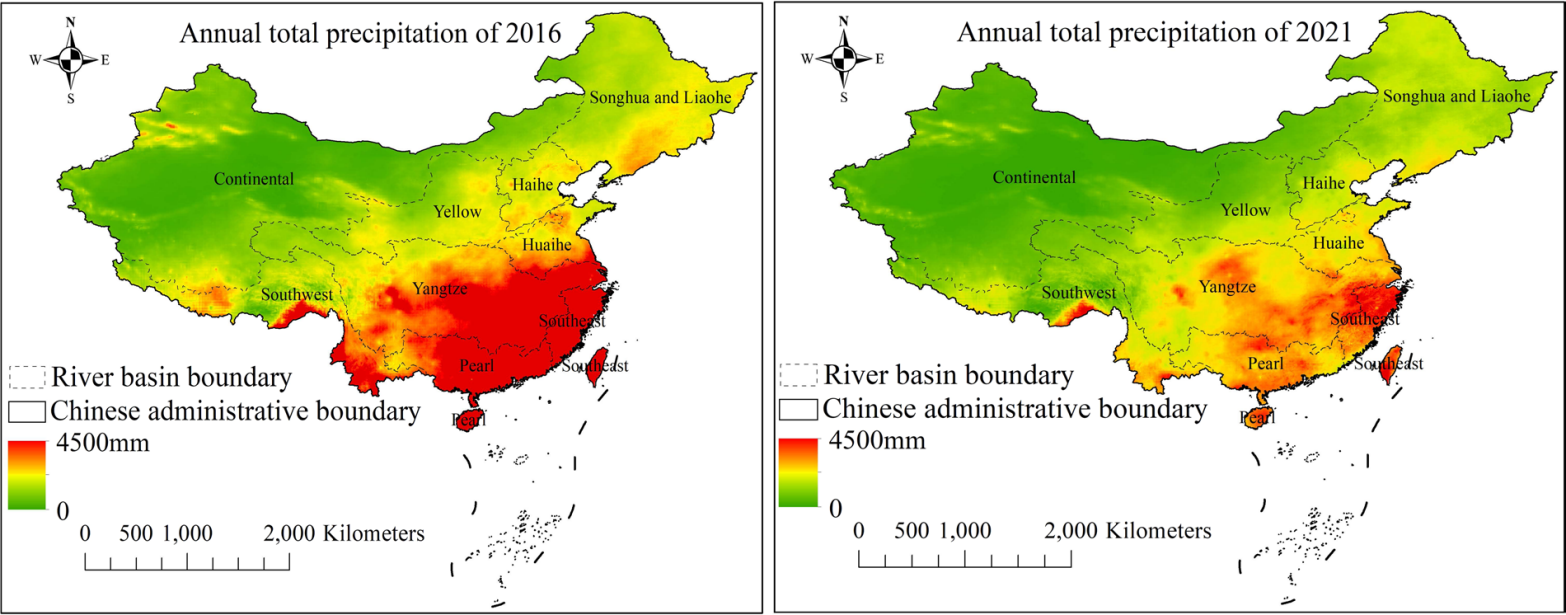
Annual accumulated precipitation pattern across China^62^ in 2016 (**a**) and 2021 (**b**).

Our study covers the entire administrative area of China. Due to its location on the west coast of the Pacific Ocean, China experiences a significant monsoon climate^33^, resulting in uneven spatial distribution of freshwater resources. The very uneven distribution of accumulated precipitation, as shown in Fig. 1, leads to severe imbalances in water resource distribution across different regions. The cultivated area in the Yangtze River Basin and south of the Yangtze River accounts for 36% of China’s total cultivated land, while its freshwater resources make up 80% of the total capacity^34^. In contrast, the water resources of the Yellow River Basin, Huaihe River Basin, and Haihe River Basin only account for 8% of the total capacity, but their cultivated land accounts for 40%^35^. In addition to the spatially imbalanced water resource distribution, interannual variation in precipitation in Northern and Southern China is another factor leading to frequent floods and droughts, resulting in greater instability for agricultural production^36^. To balance the water resources and address the aforementioned issues, the storage capacity of numerous reservoirs has been expanded to support water conservancy and irrigation for farmland, promoted by the regulation on Farmland Water Conservancy from the Chinese government. However, the efficiency of the regulation implementation remains to be explored.

### Methodological framework

Reservoir inventories are mainly achieved in two phases: water body detection and classification. In terms of water body detection, a variety of spectral band ratios have been used to adjust the threshold to distinguish water from background objects, such as snow, bare land, and vegetation, through visual interpretation^37^. The spectral band ratios include normalized difference water index (NDWI)^38^, normalized difference vegetation index (NDVI)^39^, etc. Regarding reservoir classification, visual interpretation is usually used, with the aid of Google Earth images and ancillary data^40^, to distinguish reservoir polygons from other water bodies, such as lakes.

**Fig. 2:**
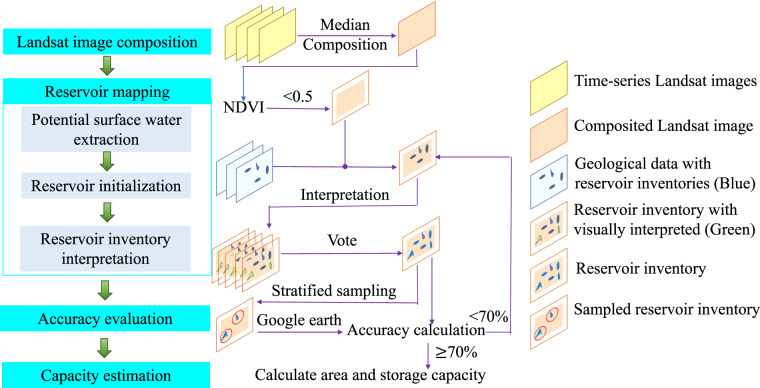
Flowchart for creating reservoir inventory.

The flow chart for creating the reservoir inventory in our study consists of four main steps: annual Landsat image composition, reservoir mapping, accuracy evaluation, and capacity estimation, as illustrated in Fig. 2.

#### Step 1: Annual landsat image composition

The annual Landsat image is composed by assigning the medium top of atmosphere (TOA) reflectance calculated from the collected annual all available Landsat7 and Landsat8 images captured during the growing season in 2016 and 2021 over China with cloud coverage of <50%. The composition generally comprises of five steps: (1) conversion from digital number of raw Landsat images to TOA^41^; (2) cloud and shadow removement by the proposed bagging model^42^; (3)radiometric correction to resist the reflectance difference of images taken in different dates triggered by different atmospheric conditions^43^; (4) calculate NDWI for each corrected Landsat image and rank the values in terms of each pixel location; (5) assign the TOA in each channel of pixel whose NDWI ranks the medium as that of corresponding composited pixel location.

#### Step 2: Reservoir mapping

Reservoir mapping involves potential surface water extraction based on spectral indices, reservoir location initialization based on geological data, and reservoir interpretation by five experts with rich experiences in dam related research. Using annually composited Landsat images of China, we first calculated the NDVI spectral index according to Eq. (1), enhancing the surface water information. The notations of I_Red_ and I_NIR_ indicate DN values of the Red and NIR bands, respectively. We set an experienced threshold of 0.5 to extract potential water bodies from background objects.

1 $${\rm{NDVI}}=\left({{\rm{I}}}_{{\rm{NIR}}}-{{\rm{I}}}_{{\rm{RED}}}\right)/\left({{\rm{I}}}_{{\rm{NIR}}}+{{\rm{I}}}_{{\rm{RED}}}\right)$$

Second, we initiated the reservoir locations based on geological data and supplemented the reservoir inventories through visual interpretation by five different experts. In cases where experts disagreed, higher spatial resolution images from Google Earth were used for further judgment. The reservoir datasets we generated had a 5-year gap, so different geological datasets were applied to initialize the reservoir locations. For the inventories of 2016, we used the Wetland Map of China and downloaded records from Baidu Maps. For the inventories of 2021, we used the National Public Basic Geographic Database and downloaded records from Baidu Maps. Additionally, we adopted the reservoir inventories generated in 2016 to initialize the 2021 inventories due to the stable attributes of reservoirs constructed over a long period of time.

With the limited spatial resolution of Landsat images, we extracted reservoirs with an area larger than 0.405 km^2^ (450 pixels × 30 m × 30 m/pixel) in the generated inventories.

#### Step 3: Mapping accuracy evaluation

The accuracy of the generated reservoir inventories in both years is evaluated using stratified sampling^44,45^ with reference to the annually composited Landsat images and higher spatial resolution images from Google Earth^46^. Five experienced experts are involved into the validation interpretation.

#### Step 4: Reservoir capacity estimation

For each mapped reservoir polygon, several attributes are added through calculations on the ArcGIS platform. These attributes include area, storage capacity, and reservoir name. Area is calculated in units of square kilometers. The storage capacity of each reservoir (*Rc*) is calculated using an experienced equation derived from work done by Yang *et al*.^7^. As illustrated in Eq. (2), the storage capacity is calculated based on area of each reservoir, indicated by notation of *Area*. The experienced equation has been validated to calculate storage capacity of 2185 reservoirs over China, referring to the statistics released by the Chinese government with R^2^ of 0.9097^7^. The strong approximate to official statistics demonstrates the strong applicability of the experienced equation in calculating storage capacity for Chinese reservoirs. Therefore, it has been adopted in our study to calculate the storage capacity for each mapped reservoir in China. Based on the storage capacity, the reservoirs were grouped into two categories: medium and large reservoirs. Medium reservoirs are defined as having a storage capacity of less than 100 million m^3^, but no less than 10 million m^3^, while large reservoirs have a capacity of no less than 100 million m^3^.

2 $$Rc=25.841\times {Area}^{1.05}$$

### Data source used for reservoirs delineation

#### Landsat images

The Landsat images used to interpret reservoirs are median composited following the methods outlined by Potapov, *et al*.^43^. The median spectral reflectance of the four spectral bands—Red, Near-Infrared (NIR), Shortwave Infrared 1 (SWIR1), and Shortwave Infrared 2 (SWIR2)—from all Landsat images throughout each year are used to determine the spectral reflectance of the annually composited image. The composited spectral reflectance is then converted to an 8-bit digital number (*DN*) using Eq. (3) for computation purposes. The values of the notation g vary in the different bands of Red, NIR, SWIR1, and SWIR2, with values of 508, 254, 363, and 423, respectively, as established by Potapov, *et al*.^43^.

3 $${\rm{DN}}={\rm{top}}\times {\rm{g}}+1$$

#### Geographical data

The geographic data used in this study include the Wetland Map of China in 2008, the National 1:250,000 Public Basic Geographic Database of 2019, and reservoir locations obtained from Baidu Maps for 2016 and 2021. Due to the coarse spatial resolution or biased local reports, most of these data sources cover only a limited number of reservoirs in China. The Wetland Map of China contains 68,521 reservoir and pond inventories, which were visually interpreted based on Landsat images from 2008 and used to initialize reservoir boundaries in China for 2016. In addition to the Wetland Map of China, the names and locations (longitude and latitude) of about 25,897 reservoirs downloaded from Baidu Maps in 2016 were used to aid in the visual interpretation of reservoir inventories.

The National Public Basic Geographic Database for 2019 is publicly available and was used to initialize reservoir inventories for 2021. It covers 197,664 inventories of surface water systems, such as lakes, reservoirs, and rivers, with reservoirs accounting for 13,020 inventories. This database is a significant public data source for initializing reservoir inventories for 2021. Additionally, the reservoir locations and names for 2021 were downloaded from Baidu Maps to aid in the visual interpretation of reservoir inventories in this study.

#### Land cover land use product

The impact of changes in reservoir capacity on irrigation cropland is analyzed using the Global Land Cover Product with Fine Classification System in 30 m (GLC_FCS30), which was proposed by Zhang *et al*.^32^. This publicly available global land use product has an overall accuracy of 82.5%^47^ and is generated from time-series Landsat images taken every 5 years from 1985 to 2020. It is classified into 29 land use categories, covering different levels of cropland, forest, shrubland, grassland, wetlands, impervious surfaces, bare areas, water bodies, and permanent ice and snow. Within the fine classification system of cropland^47^, irrigated cropland is extracted for analysis. As our study focuses on the impact of irrigation policy on reservoir storage capacity in China from 2016 to 2021, we adopt the GLC_FCS30 products for 2015 and 2020 for analysis.

### Spatial distribution pattern of medium and large and reservoirs in China

**Fig. 3:**
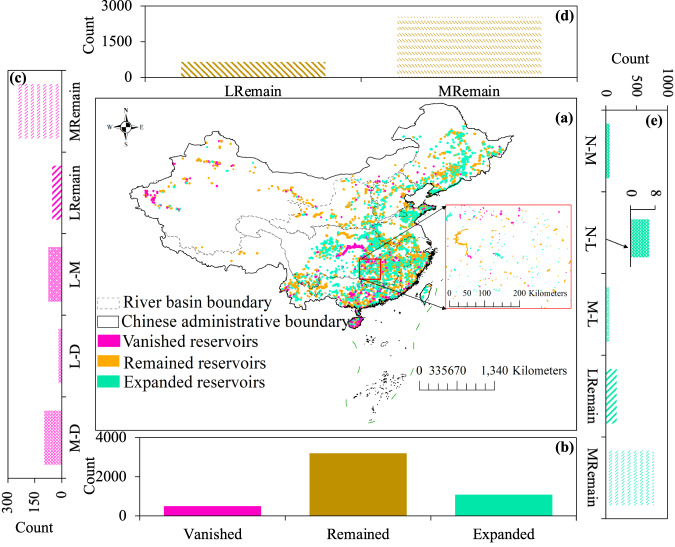
Comparison of reservoirs across China between the years 2016 and 2021. (**a**) Change in reservoir capacity from 2016 to 2021; (**b**) Number of reservoirs in different change categories (vanished, remained, expanded) from 2016 to 2021; (**c**) Vanished reservoirs with different capacity change categories from 2016 to 2021; (**d**) Remained reservoirs with different capacity sizes from 2016 to 2021; (**e**) Expanded reservoirs with different capacity change categories from 2016 to 2021. M-D and L-D represent the disappeared medium and large reservoirs, while N-M and N-L indicate newly constructed medium and large reservoirs. L-M and M-L demonstrate the category change of reservoirs from 2016 to 2021. LRemain and MRemain stand for reservoirs without category change.

In this study, aided by high-resolution images on the Google Earth platform, we utilized manual interpretation method based upon the preprocessed Landsat images on the ArcGIS 10.8.2 platform, to generate a 30 m spatial resolution reservoir inventory of China for 2016 and 2021, and calculated the corresponding attributes of reservoirs’ area and capacity, which are demonstrated in Fig. 3. The inventory was visually interpreted by five experienced experts using annually composited Landsat images after initializing them with publicly available geological datasets. The water surface area and storage capacity of reservoirs have undergone significant changes due to artificial regulation or watershed planning, to meet the continually increasing demand for hydropower energy, irrigation, flood control, drought relief, and aquaculture^48^. Since reservoir storage capacity is more widely used to indicate reservoir water supply capability^49^, storage capacity is used to distinguish the reservoirs into three categories based on the storage capacity change during the study period: expanded reservoirs, remained reservoirs, and vanished reservoirs. Expanded reservoirs are the ones with a capacity increase of more than 20% compared to that in 2016. The reservoirs whose capacity decreased by more than 20% are labeled as vanished reservoirs. The rest of the reservoirs, whose capacity changes were within 20% of that in 2016, are grouped as remained reservoirs. The 20% capacity change threshold is adopted to avoid seasonal water surface changes and boundary interpretation errors.

There are a total of 4,777 medium and large reservoirs in China from 2016 to 2021, which approximates the 4,872 reservoirs in the China water statistical yearbook. The medium and large reservoirs in 2016 had a capacity of 100,214.59 and 617,578.41 million m^3^, respectively, which is in accordance with the statistics released by the China Water Statistical Yearbook. Compared with the storage capacity in 2016, the accumulated medium and large reservoir capacity increased by 8,019.59 and 43,435.22 million m^3^ in 2021, where the storage capacity of newly constructed medium and large reservoirs contributed to 18.60% and 6.71%, respectively. With the enhanced storage capacity, the capability of water supply, flood control, and irrigation has significantly improved and been restored^50^. In 2016, the total storage capacity of medium and large reservoirs was 717,793.00 million m^3^, which was increased by 51,454.81 million m^3^ in 2021. The accumulated reservoir storage change varied significantly in different provinces and river basins of China. Most reservoirs, accounting for 66.95%, have remained the same in storage capacity, as shown in Fig. 3b. The proportion of reservoirs with expanded storage capacity, including newly extracted reservoirs, is 10.32%. The remaining reservoirs, representing about 22.73% in 2016, have vanished or disappeared until 2021. Within the vanished reservoirs (Fig. 3c), 75 large reservoirs in 2016 vanished to medium reservoirs in 2021, 120 medium and large reservoirs turned into reservoirs with a capacity of less than 10 million m^3^ or disappeared, and 298 large or medium reservoirs remained in the same category despite their vanishing storage capacity. The expanded reservoirs stem from different extents of capacity change (Fig. 3e), including 71 medium reservoirs and large reservoirs newly constructed in 2021, 57 medium reservoirs transformed to large reservoirs from 2016 to 2021, and 958 medium and large reservoirs with a remained category change.

**Fig. 4:**
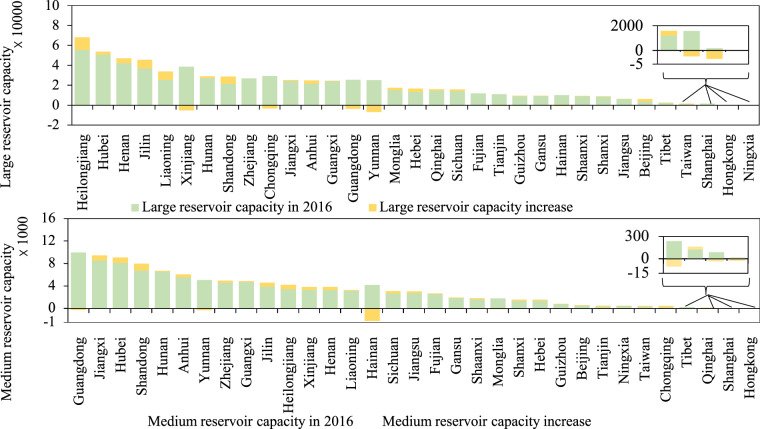
Changes in accumulated reservoir storage capacity (million m^3^) by province in China from 2016 to 2021, separated by large and medium reservoirs. The provinces are ranked from highest to lowest in terms of accumulated storage capacity.

Due to varying water use strategies and uneven precipitation patterns, the accumulated storage capacity of medium and large reservoirs across different provinces in China is significantly imbalanced. Between 2016 and 2021, most provinces have expanded their reservoir storage capacity. As shown in Fig. 4a, Heilongjiang ranks first in large reservoir capacity in 2021 with a total of 68,033.33 million m^3^, reflecting a large increase of 12,464.07 million m^3^ from 2016. In addition to Heilongjiang, three other provinces—Hubei, Henan, and Jilin—also have capacities above 40,000 million m^3^ in 2021. Notably, the storage capacity of large reservoirs in Liaoning province surpassed that of Xinjiang province, with a remarkable increase between 2016 and 2021.

The large precipitation in Liaoning province and the large water leakage resulting from the permeability of plain reservoirs in Xinjiang province^51^ are the main reasons for the change in water storage. Apart from Xinjiang province, Guangdong, Yunnan, and Chongqing provinces have lost more than 2,000 million m^3^ of water during the study period, mainly due to drought and lack of rainfall in 2021. The provinces with decreased water storage capacity require more specific and effective strategies to recover and improve their water use efficiency. In our inventory, there is no obvious large reservoir in Ningxia province that can be extracted from Landsat images.

As shown in Fig. 4b, Guangdong, Jiangxi, and Hubei provinces have the largest medium reservoir storage capacities, exceeding 9,000 million m^3^ in 2021. Guangdong province ranks first with the highest accumulated medium reservoir storage capacity throughout the study period. Shandong province witnessed the largest increase in medium reservoir storage capacity from 2016 to 2021, with 1,200.31 million m^3^, due to new reservoir construction and increased reservoir capacity implementation^6^. This corresponds with the statistics in the Jinan Water Resources Bulletin. The accumulated medium reservoir storage capacity of Hainan province decreased the most by 883.30 million m^3^, mainly due to fluctuations in annual precipitation in wet and dry years. This corresponds with the statistics in the Water Resources Bulletin of Hainan Province from 2016 to 2021. Moreover, it shows that the water conservation constructions in Hainan province require more advanced measures to improve the current relatively backward facilities to deal with fluctuating precipitation^52^.

**Fig. 5:**
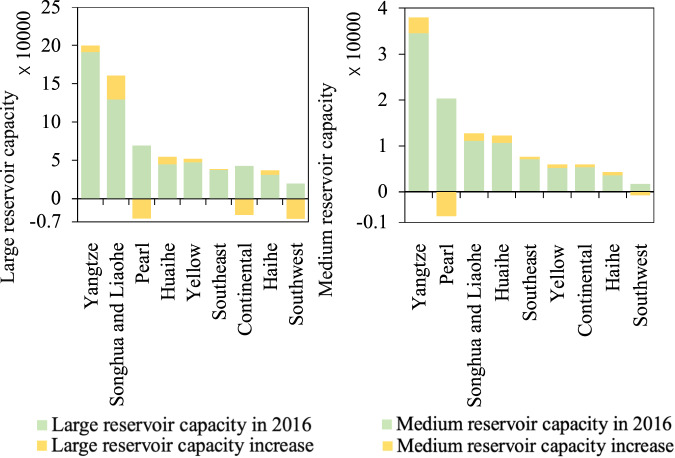
Change in accumulated reservoir storage capacity (million m^3^) in each river basin across China from 2016 to 2021. (**a**) Change in capacity of large reservoirs; (**b**) Change in capacity of medium reservoirs.

Referring to Fig. 5, most river basins in China have increased their storage capacity in both medium and large reservoirs during the study period from 2016 to 2021. The Yangtze River Basin has the highest accumulated storage capacity in both medium and large reservoirs, with proportions of 35.18% and 30.38%, respectively, in 2021. The Songhua and Liaohe River Basin expanded the largest storage capacity with 31,051.85 million m^3^ in large reservoirs of all the river basins (Fig. 5a). This is mainly due to the large increase in the accumulated capacity of Heilongjiang, Jilin, and Liaoning provinces in 2021, as shown in Fig. 4a. Due to the water leakage issue mainly in plain reservoirs in Xinjiang province, as demonstrated in Fig. 4, the accumulated storage capacity of medium reservoirs in the Continental River Basin decreased from 2016 to 2021 in Fig. 5a. The Pearl River Basin and Southwest River Basin witnessed an obvious decrease in both medium and large reservoir storage capacity during the study period, partly due to drought in Yunnan, Hainan, and Guangdong provinces.

### Use case results: reservoir storage capacity changes

**Fig. 6:**
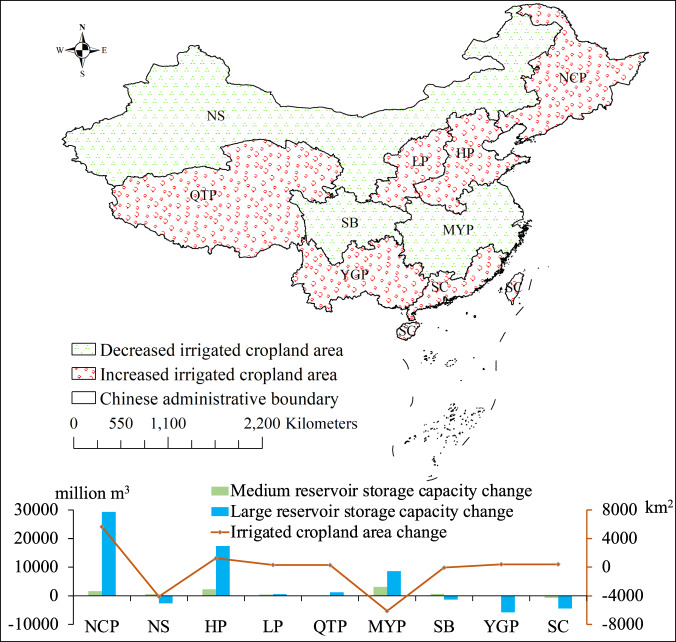
Changes in large and medium reservoir storage capacity (million m^3^) and irrigated cropland area (km^2^) for nine agricultural regions in China during the study period. (NCP: Northeast China Plain; NS: Northern arid and semiarid region; HP: Huang-Huai-Hai Plain; LP: Loess Plateau; QTP: Qinghai Tibet Plateau; MYP: Middle-lower Yangtze Plain; SB: Sichuan Basin and surrounding regions; YGP: Yunnan-Guizhou Plateau; and SC: Southern China).

We synthesized irrigated cropland products to explore the impact of changes in medium and large reservoir storage capacity on the irrigated cropland area between 2016 and 2021 in nine agricultural regions of China. As shown in Fig. 6, the accumulated reservoir storage capacity in 2016 increased with the irrigated cropland area in most agricultural regions. Notably, the accumulated large reservoir storage capacity of the Southern China and Yunnan-Guizhou Plateau agricultural regions decreased significantly, while the irrigated cropland area increased during the study period. The reason for the Southern China case mostly attributes to the droughts in 2021 and the significant improvement of water use efficiency achieved in Guangdong province through continuous optimization of water use structure^53^. It demonstrates the importance of proposing advanced strategies to improve water use efficiency and resist the impact of climatic factors such as precipitation. As for the Yunnan-Guizhou Plateau agricultural region, Yunnan province suffered from serious droughts in 2021, resulting in a remarkable decrease in storage capacity. However, the Provincial Water Resources Department in Yunnan attaches great importance to the work of saving water for farmland irrigation and places water conservation in a priority position with a series of measures, such as improving water-saving irrigation technology^54^, so that the irrigated cropland area has remained and slightly increased.

Apart from the agricultural regions with increased irrigated cropland area, the Middle-lower Yangtze Plain and Northern arid and semiarid region have significantly decreased the irrigated cropland area due to urban expansion and an increase in population for economic development^55^. Compared with the Northern arid and semiarid region, the accumulated reservoir storage capacity of the Middle-lower Yangtze Plain has increased remarkably. This is mainly owing to the extreme precipitation in Northern China in 2021^56^. With the Chinese government encouraging reservoir application for irrigation in 2016, most agricultural regions of China have witnessed an increased irrigated cropland area with enhanced water conservation capability of increased accumulated reservoir storage capacity. However, the issue of decreasing irrigated cropland area remains to be resolved with more practical solutions to avoid food crises, especially in the Middle-lower Yangtze Plain and Northern arid and semiarid region.

## Data Records

We present an inventory of medium and large reservoirs in China for the years 2016 and 2021, named (1) MLRC_2016 and (2) MLRC_2021, based mainly on Landsat 8 images and other ancillary data sources. The inventory dataset is developed by Environmental Systems Research Institute, stored in the form of a shapefile (.shp) with each record represented as a polygon and including attributes such as reservoir area, storage capacity, and reservoir name. In the attributes tables of the.shp files, the field *Shape Area* stands for the area of a reservoir polygon in square kilometers; the field *StorCap* represents the estimated storage capacity of a reservoir in million cubic meters; and the field *Name_En* refers to the name of a reservoir. The dataset gives a national medium and large reservoir extent of 23314.00 and 24888.95 km² in 2016 and 2021 respectively, with the spatial resolution of 30 m. The dataset can be accessed at repository under data doi (10.5281/zenodo.8278702)^57^.

The projected coordinate system of the dataset is as follows:

Projected Coordinate System: Krasovsky_1940_Albers

Projection: Albers;

False Easting: 0°;

False Northing: 0°;

Central Meridian: 105°E;

Standard Parallel 1: 25°N;

Standard Parallel 2: 47°N;

Latitude of Origin: 0°;

Linear Unit: Meter.

Geographic Coordinate System: GCS_Krasovsky_1940

Datum: D Krasovsky 1940;

Prime Meridian: Greenwich;

Angular Unit: Degree.

## Technical Validation

In order to evaluate the comprehensiveness and accuracy, we compared the proposed reservoir inventory MLRC_2016 and MLRC_2021 with the publicly accessible reservoir datasets covering China in terms of large and medium reservoir number, area, storage capacity, the number of reservoirs with attribute of names, and acquisition year. The publicly accessible reservoir datasets comprise of China-LDRL^29^, CRD database^6^, GOODD^22^, GeoDAR v1.1^23^, GRanD v1.3^2^, National 1:250000 Public Basic Geographic Database of 2019 (ND2019) (https://www.webmap.cn/main.do?method=index), and the recently published Global Reservoir Storage (GRS) dataset^58^, as listed in Table 1. It is challenging to conduct a fair comparison between our two-phase inventory of 2016 and 2021 and the published reservoir datasets, because the publicly released datasets are generally collected from images and statistics of different years than our proposed MLRC dataset (Table 1). Since actual reservoir mapping over China is limited accessible, the publicly released census of China water statistical yearbook (CWY) of year 2016 and 2021 are referred to as ground truth in this study with actual statistics of reservoir number and storage capacity. Detailed comparisons are listed in Table 1. The reservoirs of the inventories for comparison are subtracted to Chinese administrative area with the storage capacity of no less than 10 million m^3^, in accordance with the focus of our reservoir inventory on medium and large reservoirs.

**Table 1:** Statistical comparison between our proposed reservoir inventory with publicly available datasets, in terms of reservoir number, coverage area(km^2^), storage capacity (million m^3^), number of reservoirs with names, and acquisition year.

Reservoir Dataset	Acquisition year	Number of reservoirs	Coverage Area	Storage Capacity	Number of reservoirs with names
China-LDRL	2019	2194	16351.95		
ND2019	2015–2017	6728	21432.57		6728
CRD	1984–2020	885	28211.08	79569.41	856
GOODD	2020	9215 dams			
GRanD	2019	885	16844.41		
GeoDAR	2019	1713	18162.84		
GRS	1999–2018	885			
MLRC	2016	4631	23314.00	717793.00	3862
	2021	4602	24888.95	769247.81	4071
CWY	2016	4610		826200	
	2020	4872		858000	

It is apparent that our proposed reservoir inventory draws closest to the actual number of reservoirs and storage capacity in both years of 2016 and 2021, as listed in the census of China water statistical yearbook. The dataset of GOODD only comprises of locations of dams, rather than detailed inventories, lacking detailed reservoir spatial characteristics. ND2019 is a composite dataset with of 6728 reservoir inventories, which is far larger than the actual reservoir number, indicating many falsely extracted reservoirs in the ND2019 dataset. The datasets of China-LDRL and GeoDAR in China are captured in year of 2019, which should cover more reservoirs that that of year 2016, but the number of their recorded reservoirs is far smaller than that of China water statistical yearbook in year of 2016. In terms of CRD and GRanD datasets, the numbers of reservoirs are both 885, indicating large quantities of large and medium reservoirs are missed in the datasets. It needs to point the dataset GRS provides monthly reservoir statistics, but the detailed inventory is inaccessible publicly and the inventory shown on the platform of Google Earth Engine for each reservoir does not change over time as well. With the limited number of reservoirs recorded in China in GRS, the application of GRS dataset is largely restricted. Referring to the census of China water statistical yearbook, our proposed MLRC dataset is recognized as most reliable with the most approximate number of reservoirs and storage capacity.

**Fig. 7:**
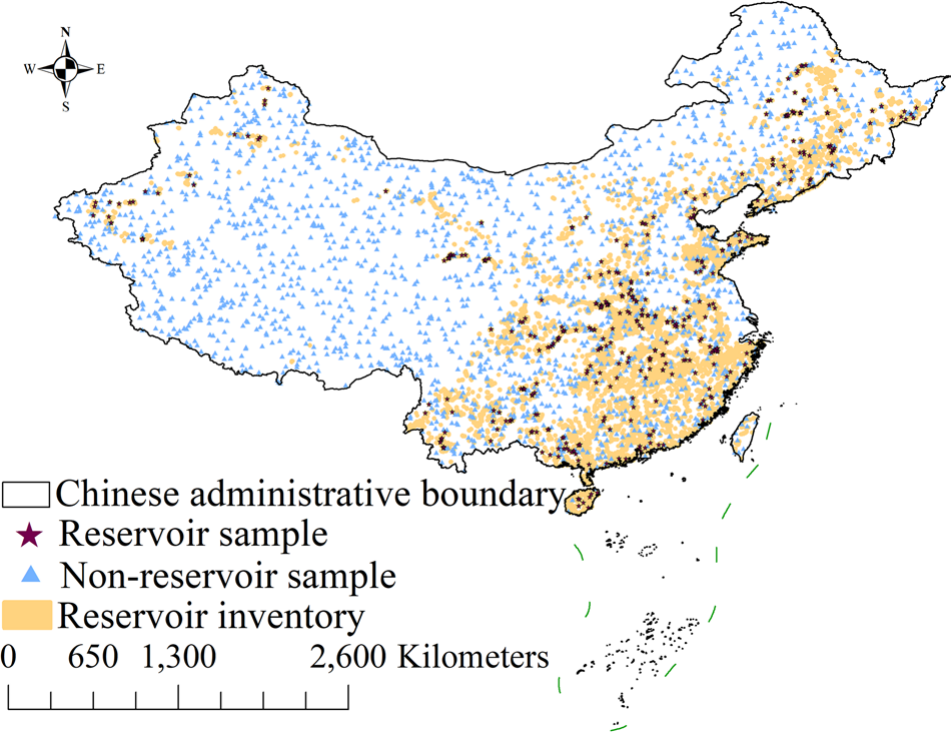
Evaluation sample distributions of reservoir and non-reservoir ground objects.

**Fig. 8:**
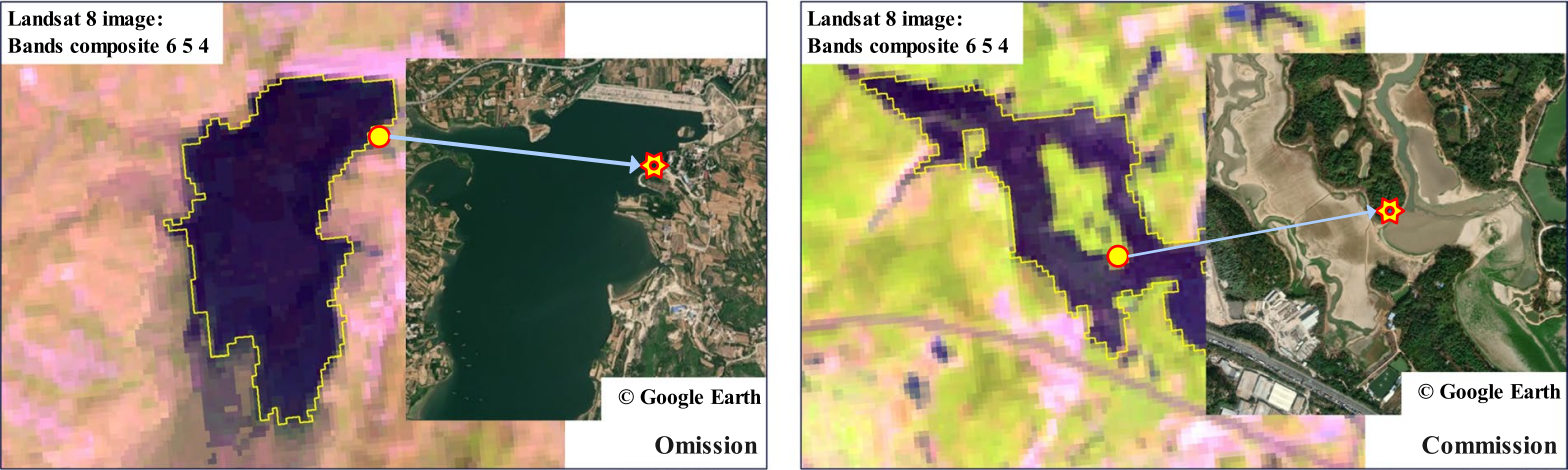
Demonstration of omitted and committed reservoir samples with reference to high spatial resolution images from Google Earth. (**a**) Example of omitted actual reservoir pixel; (**b**) Example of committed actual background pixel but mis-extracted as a reservoir.

To present an objective evaluation, our inventory is evaluated by visual interpretation with the strategy of stratified sampling^44,45^. Stratified sampling is an evaluation strategy that has been widely used for large-scale object mapping evaluation^59^. The reservoir inventories in MLRC_2016 and MLRC_2021 are evaluated using selected samples, as shown in Fig. 7. We randomly selected 400 reservoir sample points within the inventory and 2,000 non-reservoir sample points outside the inventory. Referring to annually composited Landsat images and high-resolution images from Google Earth, the selected reservoir and non-reservoir samples are validated through visual interpretation by five experienced experts and voted in case of disagreement. We calculated the indicators of user accuracy (UA), producer accuracy (PA), and overall accuracy (OA) to objectively evaluate the accuracy of our proposed inventory. UA is used to measure the percentage of correctly extracted reservoir samples. PA is used to evaluate the percentage of correctly extracted reservoir samples over all the actual reservoir samples. OA is a comprehensive indicator of both accuracies. The inventories of reservoirs are mostly accurate, with an OA of 99.71%, as listed in Table 2. Most extracted reservoir pixels are correct, with a 99.50% UA, and most actual reservoir pixels have been correctly extracted with a 98.75% PA in our inventory as well. Due to the limit of Landsat image resolution and pixel mixture issue^60^, some reservoir points were omitted and committed as background objects on the boundary of the reservoirs, as referred to high-resolution Google Earth images in Fig. 8. This issue can be further addressed by adopting a mixed pixel decomposition strategy^61^ in future studies.

**Table 2:** Reservoir inventory proposal evaluation statistics.

Category	Reservoir	Background	UA (%)	PA (%)	OA (%)
Reference reservoir	395	2	99.5014	98.7514	99.71
Reference background	5	1998	99.75	99.90

## Usage Notes

By distinguishing the reservoirs based on uniform data sources and methodology, the proposed reservoir inventory dataset is intended for further analysis to explore the impact of anthropogenic activities and climate change on changes in China’s water landscape. Additionally, it can be used to investigate the effects of changes in reservoir storage capacity on geological and hydrological hazards. This detailed reservoir inventory provides a fundamental data source to develop a more effective plan for allocating limited water resources in China. And it is believed to be useful for promoting the research of basic disciplines such as hydrology and environmental science, and assisting watershed-scale environmental planning/monitoring and the national macro-decision-making in terms of integrated management of surface water resources.

In addition, unlike current studies that present single temporal inventories, our study proposes a consistent inventory for two different years, allowing the observation of variations in reservoir construction. In response to the regulations on Farmland Water Conservancy proposed by the Chinese government in 2016, which encourage the application of reservoirs for irrigation, our two-phase reservoir inventory has shown the remarkable positive impact of increased accumulated reservoir storage capacity on the expansion of irrigation cropland area. In the face of uncontrollable climatic factors, particularly extreme precipitation or droughts, proposing advanced strategies for higher water use efficiency is more significant in guaranteeing water use. Compared with a single temporal reservoir inventory, the proposed two-phase reservoir inventory can contribute to providing more insight analysis of a wide range of water conservation management-related aspects for better sustainable development.

**Fig. 9:**
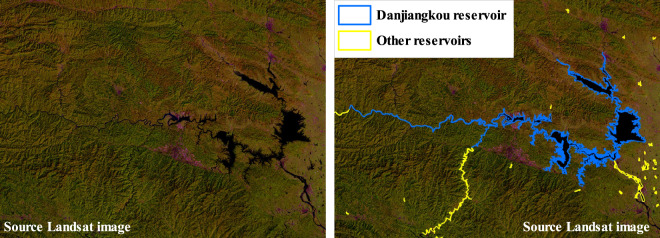
Demonstration of Danjiangkou reservoir.

The inventories in this study cover medium and large reservoirs, which can be easily confused with large rivers or lakes, especially those located closely. Danjiangkou reservoir, spanning across Hubei and Henan provinces, is a typical reservoir constructed beside the Yangtze River (as shown in Fig. 9). The starting location is easy to recognize in the annually composited Landsat image, but the ending boundary is difficult to extract compared to closed reservoirs. In this study, the ending boundary of such reservoirs without exact closed boundaries is extracted by referring to the official census released from the yearbook through calculating reservoir area and storage capacity and the relative location to nearby reservoirs. Moreover, due to the issue of mixed pixels in the Landsat image, as demonstrated in Fig. 8, the extracted boundaries of reservoirs are difficult to delineate the detailed shapes. This issue can be addressed by improving the inventory based on higher spatial resolution images in future studies.

The names provided in the dataset for each reservoir are collected from public geological datasets and local media, but there are still several names of reservoirs missing. These can be filled or updated by local field survey, which can be time-consuming and expensive to implement. To the best of our knowledge, the names provided in our proposed inventory cover the state-of-the-art largest coverage. This can be recognized as a comprehensive and reliable reservoir dataset for hydrological modeling and improving water management efficiency.

## Data Availability

The code for extracting names, longitudes and latitudes of reservoirs, ponds, etc. from Baidu web map (http://map.baidu.com) can be obtained from https://github.com/lidc54/webMap. The annual Landsat image composition is implemented on the platform of Google Earth Engine. One of the main tools to visualize the dataset is to use the software ArcGIS Desktop see https://desktop.arcgis.com/en/arcmap/latest/get-started/installation-guide/installing-on-your-computer.htm. By using ArcGIS 10.8.2 platform, we distinguished and vectorized the reservoirs’ boundaries based on manual interpretation on Landsat 8 images.
